# Sustained therapeutic effect of an anti-inflammatory peptide encapsulated in nanoparticles on ocular vascular leakage in diabetic retinopathy

**DOI:** 10.3389/fcell.2022.1049678

**Published:** 2022-12-16

**Authors:** Qiang Qu, Kyoungmin Park, Kevin Zhou, Drew Wassel, Rafal Farjo, Tracy Criswell, Jian-xing Ma, Yuanyuan Zhang

**Affiliations:** ^1^ Department of Ophthalmology, The First Affiliated Hospital of Fujian Medical University, Fuzhou, China; ^2^ Joslin Diabetes Center, Harvard Medical School, Boston, MA, United States; ^3^ Department of Biochemistry, Wake Forest University School of Medicine, Winston-Salem, NC, United States; ^4^ EyeCro LLC., Oklahoma City, OK, United States; ^5^ Institure for Regenerative Medicine, Wake Forest University School of Medicine, Winston-Salem, NC, United States

**Keywords:** pigment epithelium-derived factor, anti-inflammatory, anti-vascular leakage, diabetic retinopathy, wnt signaling, PLGA, nanoparticles

## Abstract

Pigment epithelium-derived factor (PEDF), an endogenous Wnt signaling inhibitor in the serine proteinase inhibitors (SERPIN) super family, is present in multiple organs, including the vitreous. Significantly low levels of PEDF in the vitreous are found to associate with pathological retinal vascular leakage and inflammation in diabetic retinopathy (DR). Intravitreal delivery of PEDF represents a promising therapeutic approach for DR. However, PEDF has a short half-life after intravitreal injection, which represents a major hurdle for the long-term treatment. Here we report the prolonged therapeutic effects of a 34-mer peptide of the PEDF N-terminus, encapsulated in poly (lactic-co-glycolic acid) (PLGA) nanoparticles (PEDF34-NP), on DR. PEDF34-NP inhibited hypoxia-induced expression of vascular endothelial growth factor and reduced levels of intercellular adhesion molecule 1 (ICAM-1) in cultured retinal cells. In addition, PEDF34-NP significantly ameliorated ischemia-induced retinal neovascularization in the oxygen-induced retinopathy rat model, and significantly reduced retinal vascular leakage and inflammation in streptozotocin-induced diabetic rats up to 4 weeks after intravitreal injection, as compared to PLGA-NP control. Intravitreal injection of PEDF34-NP did not display any detectable toxicities to retinal structure and function. Our findings suggest that PEDF34-NP can confer sustained therapeutic effects on retinal inflammation and vascular leakage, having considerable potential to provide long-term treatment options for DR.

## 1 Introduction

Diabetic retinopathy (DR) as a major complication of diabetes mellitus is the leading cause of blindness in the working-age population (Klein et al., 1995; Aiello et al., 1998; Gardner et al., 2002). Retinal inflammation and the breakdown of the blood-retinal barrier (BRB) play causative roles in the development of diabetic macular edema (DME) in DR (Bhagat et al., 2009; Tang and Kern, 2011). In proliferative diabetic retinopathy (PDR), retinal neovascularization, aberrant new blood vessels in the retina can further lead to elevated risks of retinal hemorrhage and detachment (Aiello et al., 1998; Adamis et al., 1999; Gardner et al., 2002). A main therapeutic approach to prevent visual impairment in DR is laser-induced photocoagulation, decreasing the incidence of blindness from PDR by 95% (Ahmadi and Lim, 2009; Bhagat et al., 2009). However, there are potential adverse effects such as loss of visual acuity, night blindness, and accidental burns in the fovea (Fong et al., 2007).

Vascular endothelial growth factor (VEGF) as the pro-angiogenic factor prompts the tissue regeneration, however, it facilitates the development and progression of DME and PDR (Adamis et al., 1994; Aiello et al., 1994; Adamis et al., 1996; Aiello, 1996; Auricchio et al., 2002). Clinically, drugs targeting VEGF have shown impressive efficacy in the treatment of DME and neovascularization in DR patients (Stewart, 2016; Cai and Bressler, 2017). However, approximately 40% DR patients do not respond to anti-VEGF drugs, suggesting other pathogenic factors are involved in the vitreous (Adamis et al., 1994; Aiello et al., 1994; Adamis et al., 1996; Aiello, 1996; Auricchio et al., 2002). Further, anti-VEGF treatment has been reported to associate with retinal fibrosis and neurodegeneration (Ahn et al., 2016; Tan et al., 2021; Xiao et al., 2021). Therefore, novel therapeutic modalities localized within the vitreous are highly desired.

Pigment epithelium-derived factor (PEDF) as an endogenous anti-angiogenic and anti-inflammatory factor in the serine proteinase inhibitors (SERPIN) family (Dawson et al., 1999; Bouck, 2002; Tombran-Tink and Barnstable, 2003b) is expressed in multiple tissues and organs, including vitreous (He et al., 2015). PEDF does not affect the function and structure of existing vascular system (Yang et al., 2016); but it inhibits pathological angiogenesis induced by oxidative stress, ischemia or tumor toxicant-induced inflammation (He et al., 2015). However, the levels of PEDF in retina and vitreous tissues are significantly reduced in DR patients and animal models (Ogata et al., 2001; Boehm et al., 2003; Gao et al., 2001). The decreased expression of endogenous anti-pathologically angiogenic factors such as PEDF in addition to the overexpression of pro-angiogenic factors such as VEGF have been shown to disturb the angiogenic balance and are responsible for the retinal vascular leakage and neovascularization (King and Suzuma, 2000; Gao et al., 2001; Saky et al., 2001; Gao et al., 2002). In addition to its anti-angiogenic activities, PEDF functions to decrease vaso-permeability, inflammation and fibrosis (Tombran-Tink and Barnstable, 2003b; Eichler et al., 2004; Zhang et al., 2005b; Zhang et al., 2006). Delivery of the PEDF gene using adenovirus (Rasmussen et al., 2001), adeno-associated virus, synthesized peptide or plasmid significantly suppressed ischemia-induced retinal neovascularization in oxygen-induced retinopathy (OIR) (Raisler et al., 2002; Barnstable and Tombran-Tink, 2004; Thumann et al., 2017). In 2005, it was demonstrated that a 34mer (amino acid 44–77) peptide of PEDF (PEDF34) displayed an antitumor effect in prostate cancer cells (Filleur et al., 2005; Filleur et al., 2009). Another group reported that PEDF34 also inhibited choroidal neovascularization, suggesting that this 34mer peptide enhances anti-angiogenic activity (Filleur et al., 2005; Amaral and Becerra, 2010).

Despite the effective therapeutic potential, PEDF application is limited due to its short half-life, unstable pharmacology activity, and administration pathway (Bai et al., 2012). As repeated intravitreal injections are not ideal, a novel system allowing for sustained and long-term release of PEDF34 in the vitreous is needed, one of these sustained-release delivery vehicles for ocular drugs is biodegradable poly (lactic-co-glycolic acid) (PLGA) nanoparticles (NP). NPs have been used as sustained delivery vehicles for drugs or biological materials due to 1) the potential to design sustained release of a peptide resulting in long-term efficacy, reduced frequency of intravitreal injections, lower cost, and reduced injection-related systemic side effects (Busik et al., 2009); 2) a high concentration of the peptide can be loaded in the NP, resulting in longer intraocular retention (Abramson et al., 2013); and 3) the small particle size can be consistently delivered *via* local injection.

Recently published reports explored the advances or effective role of NPs in neurological disorders (Sharma et al., 2022a; Sharma et al., 2022b; Rhaman et al., 2022; Shohag et al., 2022). Our investigation intend to develop a sustained therapeutic system of an anti-inflammatory peptide encapsulated in NPs to reduce ocular vascular leakage in the prevention and treatment of DR. Thus, we evaluated the efficacy of the prolonged intravitreal delivery of PEDF34mer peptide using PLGA NP (PEDF34-NP) on retinal inflammation, vascular leakage in streptozotocin (STZ)-induced diabetic model and on retinal neovascularization in the OIR model.

## 2 Materials and methods

### 2.1 Cell culture

Primary bovine retinal capillary endothelial cells (BRCEC) were isolated from bovine eyes and cultured following a documented procedure (Zhang et al., 2001). Cells at passages 4 and 5 were used for this project.

ARPE19 cells, a cell line derived from human retinal pigment epithelial (RPE) cells, were purchased from American Type Culture Collection (ATCC, Manassas, VA). ARPE19 cells were cultured in Dulbecco’s Modified Eagle Medium (DMEM) containing 10% fetal bovine serum (FBS). Primary BRCEC were cultured in DMEM containing 10% FBS and 1% insulin transferrin selenium. All the common cell culture materials were purchased from Cellgro (Manassas, VA). Cultured cells were fasted in DMEM containing 1% FBS for 4 h before addition of the proteins or compounds.

### 2.2 Preparation of PEDF34-PLGA nanoparticles

The PEDF34-PLGA nanoparticles (PEDF-PLGA-NP) were prepared using a water-oil-water double emulsion method as previously described with modifications (Jin et al., 2011). Briefly, 10 mg of the PEDF34 peptide was dissolved in 200 μL ultrapure water, and the PEDF34 peptide solution was then added into the organic phase composed by 100 mg of PLGA dissolved in 2 ml dichloromethane to form the primary emulsion by using an ultrasonic processor at 5% amplitude for 1 min in an ice bath. The primary emulsion was mixed with a 0.8% weight/volume (w/v) F68 aqueous solution and emulsified by sonication at 10% amplitude for 5 min (min) in an ice bath. Then, the resulting double (w/o/w) emulsion was left stirring. The PEDF34-PLGA-NP were collected in the supernatant by centrifugation at 3,000 rpm for 5 min at 4°C. The nanoparticle formulation exhibited diameter of 160 ± 20 nm, Unencapsulated peptide was washed off. The blank PLGA nanoparticles were prepared without PEDF34.

### 2.3 Western blot analysis

For *in vitro* assays, cells were washed with cold phosphate buffered saline (PBS) and lysed in radioimmunoprecipitation assay (RIPA) buffer (150 μM [mM] NaCl, 1% NP-40, 0.1% sodium dodecyl sulfate (SDS), 50 mM Tris-HCl, pH 8.0, 1 mM ethylenediaminetetraacetic acid (EDTA), and 1 mM phenylmethylsulfonyl fluoride (PMSF). For *in vivo* assays, the retinas were carefully dissected from rat eyes. Retina homogenates were prepared in 0.1% SDS extraction buffer containing 50 mM Tris-HCl, pH 7.8, 5 mM EDTA, 1% NP-40, 2.5% glycerol, 100 mM NaCl and 1 mM fresh PMSF. The homogenates were cleared by centrifugation at 12,000 × *g* for 20 min at 4°C, and the protein concentration was determined using the Bradford assay. Retinal proteins (50 µg) were resolved by 10% SDS-polyacrylamide gel electrophoresis (SDS-PAGE) and transferred onto nitrocellulose membranes. The membrane was blocked with 10% non-fatty milk in tris-buffered saline with 0.1% Tween 20 (TBST) for 2 h. Rabbit polyclonal antibodies against intercellular adhesion molecule 1 (ICAM-1) (1:1,000 in 5% milk, v/v) and VEGF (1:3,000 in 5% milk, v/v) as well as a goat polyclonal anti-connective tissue growth factor (CTGF) antibody (1:100 in PBS, v/v) were purchased from Santa Cruz Biotechnology, Inc. (Santa Cruz, CA). Rabbit polyclonal antibody against tumor necrotic factor-alpha (TNF-α, 1:1,000 in 5% milk, v/v) was purchased from Abcam (Cambridge, MA). The goat polyclonal antibody for albumin (1:5,000 in 5% milk, v/v) was purchased from Bethyl Laboratories Inc. (Montgomery, TX). Chemiluminescent membranes were washed and incubated with horseradish peroxidase (HRP)–tagged goat anti-rabbit immunoglobulin M (IgM), HRP-tagged goat anti-mouse IgG, or HRP-tagged goat anti-rabbit IgG and visualized (Vector Laboratories, Burlingame, CA). For the quantification of the signal intensity, the densitometry was performed using SynGene tool imager software (SynGene, Frederick, MD).

### 2.4 Cell viability assay

Viable cells were quantified by 3-(4,5-dimethylthiazol-2-yl)-2.5-diphenyl-2H-tetrazolium bromide (MTT) assay (Roche, Indianapolis, IN) as described previously. Briefly, buffer^#^1 (i.e., MTT), 10% volume of the culture medium) was added to the cell culture after desired treatments and incubated for 4 h. Then 100% culture volume of buffer^#^ 2 (i.e., 10% SDS in 0.01 M HCl) was added and incubated with the cells for 16 h. Optical absorbance at 570 nm was measured using an enzyme-linked immunosorbent assay (ELISA) reader.

### 2.5 Oxygen-induced retinopathy

Care and use of all animals in this study were in strict agreement with the guidelines in the Care and Use of Laboratory Animals set forth by the University of Oklahoma. Brown Norway rats from Charles River Laboratories (Wilmington, MA) were used for OIR model following an established protocol (Smith et al., 1994). Newborn rats (along with their nursing mothers) were exposed to 75% oxygen from postnatal day 7 (P7) to P12 in a Plexiglass chamber connected to an oxygen regulator (Pro-Ox, model 110; Reming Bioinstruments, Redfield, NY). At P12, the rats were returned to room air. The animals were anesthetized with intraperitoneally injected with 50 mg/kg ketamine hydrochloride and 5 mg/kg xylazine (Vedco, St. Joseph, MO) and received an intravitreal injection of 20 µg PEDF34-NP through a 32-gauge needle (Hamilton Co., Reno, NV) under a dissecting microscope, with the same amount of empty PGLA-NP control served as the control group. The rats were euthanized at P16 for Western blot analysis and permeability assay or sacrificed at P18 for retinal neovascularization examination.

### 2.6 Rat models of streptozotocin-induced diabetes

Two-month-old Brown Norway rats weighing approximately 200 g (g) were obtained from Charles River Laboratories (Wilmington, MA). All the animals were treated according to the ARVO Statement for the Use of Animals in Ophthalmology and Vision Research. After 12 h (hr) fasting, the animals received a single 50 mg/kg intraperitoneal (IP) injection of STZ (Sigma-Aldrich, St. Louis, MO) in 10 mM of sodium citrate buffer, pH 4.5. Control (non-diabetic) animals were fasted and injected with the citrate buffer alone. Animals with blood glucose levels greater than 300 mg/dl were considered diabetic and randomly divided into groups. Diabetic rats at 2 weeks after onset of diabetes received an intravitreal injection of PEDF34-NP (20 µg/eye) in the treatment group and the same numbers of empty PGLA-NP (Control-NP) in the control group.

### 2.7 Retinal fluorescein angiography and pre-retinal vascular nuclei quantification

At P18 of age, rats received an intraperitoneal injection of an anesthetic cocktail, containing ketamine, xylazine and acepromazine. The rats were then perfused through the left ventricle with a solution of 1 ml PBS containing 50 mg high molecular–weight (2 × 10^6^) fluorescein isothiocyanate (FITC)-conjugated dextran (Sigma-Aldrich, St. Louis, MO). The eyes were enucleated and fixed in 4% paraformaldehyde (PFA) for 3 h at 4°C. The lens was removed, and the retina was incised radially. The retina was flat mounted with a mounting medium (Vector Laboratories, Burlingame, CA). Retinal vessels were visualized by fluorescence microscopy and photographed with a Leica SP2 MP Confocal microscope (Leica, Bannockburn, IL). The non-perfusion area was measured in the retina using SPOT software (Diagnostic Instruments, Sterling Heights, MI).

For quantification of pre-retinal neovascularization, the eyes were sectioned and stained with hematoxylin and eosin (H&E). Pre-retinal vascular nuclei (anterior to the inner limiting membrane) were counted in a masked fashion following a documented procedure (Smith et al., 1994).

### 2.8 Real-time reverse transcription–polymerase chain reaction

Total RNA was extracted from eyecups using TRIzol reagent (Invitrogen, Carlsbad, CA). The complementary deoxyribonucleic acid (cDNA) was synthesized from 1 μg of total ribonucleic acid (RNA) using TaqMan® Reverse Transcription Reagents according to the manufacturer’s protocol (Applied Biosystems, Foster City, CA), and the reaction product was subjected to real-time PCR amplification using a MyiQ Bio-rad thermal cycler (Bio-rad, Hercules, CA). The following primers were used for the PCR: for VEGF, 5′-ATG​AAC​TTT​CTG​CTG​TCT​TGG​GTG-3′ (forward) and 5′-TCA​CCG​CCT​CGG​CTT​GTC​ACA-3′ (reverse); for CTGF, 5′-GTT​GTT​CAT​TAG​CGC​ACA​GTG-3′ (forward) and 5′-TAG​AGC​AGG​TCT​GTC​AAG​CAT-3′ (reverse); for ICAM-1, 5′-TGC​GTT​TTG​GAG​CTA​GCG​GAC​CA-3′ (forward) and 5′-CGA​GGA​CCA​TAC​AGC​ACG​TGC​CAG-3′ (reverse); for TNF-α, 5′-ACA​AGG​CTG​CCC​CGA​CTA​T-3′ and 5′-CTCCTGGTATGAAGTGGCAAATC-3′(reverse); and for Glyceraldehyde-3-Phosphate Dehydrogenase (GAPDH), 5′-ATG​GTG​AAG​GTC​GGT​GTG​AAC-3′ (forward) and 5′-GTG​CCG​TTG​AAT​TTG​CCG​TGA-3′ (reverse).

### 2.9 Measurement of retinal vascular permeability

Retinal vascular permeability was measured using the Evans blue-albumin leakage assay as described previously (Zhang et al., 2005a). Briefly, animals were anesthetized and retinal vascular permeability was measured using Evans blue dye (Sigma-Aldrich, St. Louis, MO) injected through the tail vein over 10 s at a dosage of 45 mg/kg. After 2 h, the rats were perfused for 10 min at a physiological pressure *via* the left ventricle with citrate-buffered PFA. The perfusion solution was warmed to 37°C to prevent vasoconstriction. Immediately after perfusion, both eyes were enucleated and bisected at the equator. The retinas were carefully dissected, and Evans blue was extracted by incubating with formamide for 18 h at 70°C. The supernatant was harvested through ultracentrifugation at 70,000 x*g* for 2 h. The concentration of the dye in the extracts was calculated from a standard curve of Evans blue in formamide. Concentrations of Evans blue in the retina were normalized by total retinal protein concentrations.

### 2.10 Electroretinogram recording

For scotopic electroretinogram (ERG), rats were dark-adapted overnight prior to ERG recording using Espion E3 system Ganzfeld Color Dome system (Diagnosys LLC, Lowell, MA). The rats were anesthetized, and the pupils dilated with topical application of 2.5% phenylephrine and 1% tropicamide. ERG responses were recorded with a silver chloride needle electrode placed on the surface of the cornea after topical application of 1% tetracaine anesthesia. A reference electrode was positioned at the nasal fornix, and a ground electrode on the tail. Body temperature was maintained at about 37°C with a heating pad. The ERG system consisted of a Ganzfeld bowl, a DC amplifier, a computer-based control and a recording unit. ERG responses were recorded in response to brief (<1 ms.) white light flashes from the Xenon arc delivered at 10mSec intervals for dim stimuli and for intervals up to 60 s for high-intensity flashes. The band-pass filter cutoff frequencies were 40 and 200 Hz for single-flash or flicker-stimulus recordings, respectively. Single-flash responses were recorded under both dark-adapted (scotopic) and light-adapted (photopic) conditions. The range of stimulus intensities extended from 1.6 × 10^−5^ to 11 cd s/m^2^, and each data point was derived from the average of three recordings. Fourteen scotopic responses were recorded and averaged, with flash intervals of 10 mSecs. Immediately after scotopic ERG, rats were exposed to a background light of 60 cd m^−2^ for 10 min. Photopic ERG was then recorded by averaging the responses to 14 single flashes.

### 2.11 Statistical analysis

All values are expressed as mean ± standard deviation (SD). The results were analyzed by statistical analysis using the Student’s *t* test (two-tailed) for comparison of two groups and ANOVA for comparison of multiple groups. A value of *p* < 0.05 was pre-determined as the criterion of significance.

## 3 Results

### 3.1 Inhibitory effects of PEDF34-NP on vascular endothelial growth factor expression and endothelial cell growth under hypoxia

**FIGURE 1:**
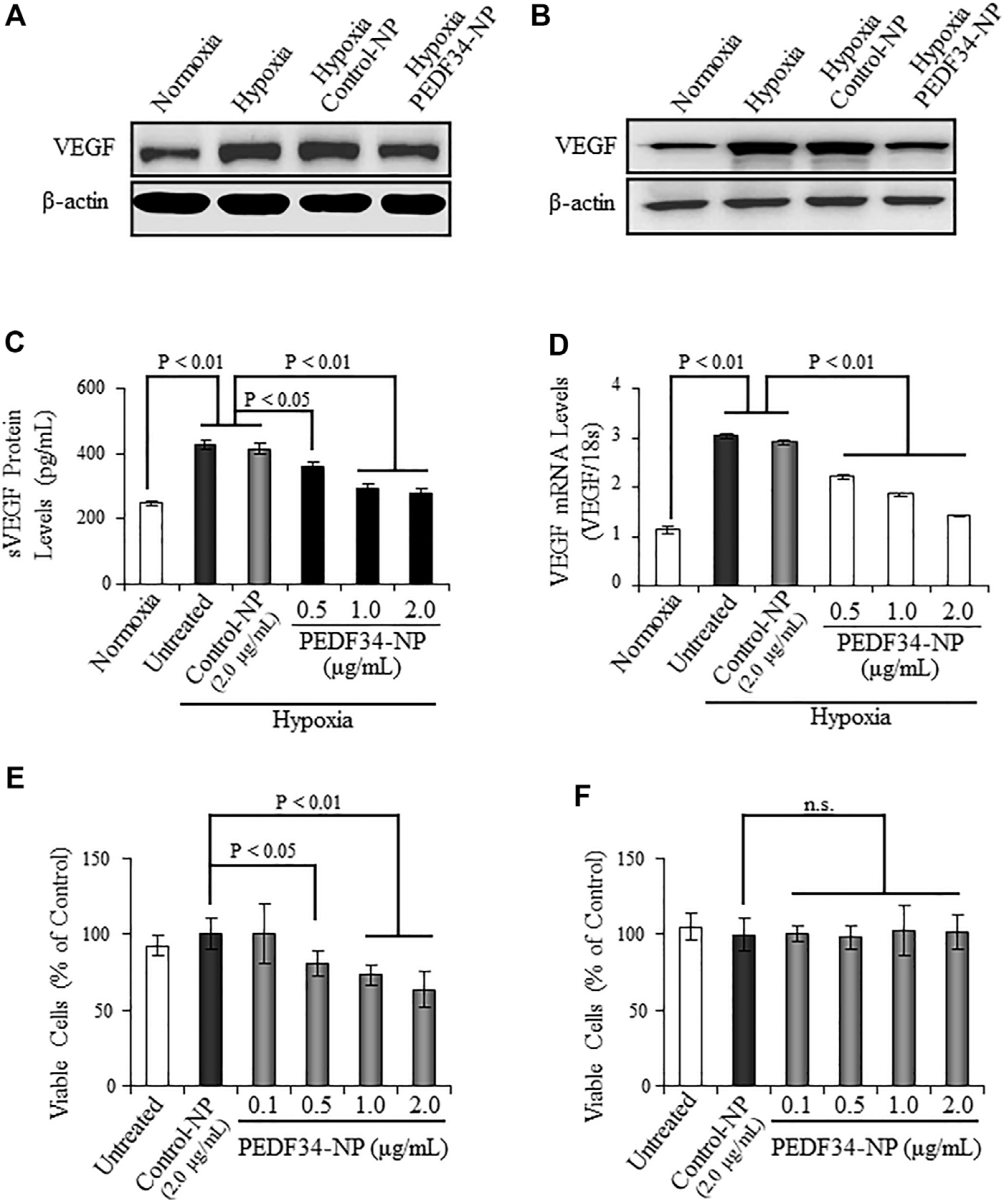
Inhibitory effect of PEDF34-NP on VEGF expression induced by hypoxia in ARPE19 cells and BRCEC. **(A)** ARPE19 cells were grown to 70% confluence in media containing 2% FBS and treated with PEDF34-NP at 1 μg/ml for 16 h under hypoxia (1% oxygen). The same amount of total cellular proteins (30 µg) was applied for Western blot analysis using the antibody specific for VEGF. **(B)** BRCEC were exposed to hypoxia and PEDF-34NP for 16 h. VEGF levels in the cells were measured by Western blot analysis. **(C)** and **(D)** ARPE19 cells were treated with Control-NP and various concentrations of PEDF34-NP for 24 h under hypoxia. VEGF secreted into the media (sVEGF) was measured by ELISA using an ELISA kit **(C)** and VEGF mRNA levels were measured by real-time RT-PCR and normalized by 18S rRNA levels **(D)**. **(E)** and **(F)** BRCEC **(E)** and ARPE19 cells **(F)** were treated with PEDF34-NP at the indicated concentrations for 24 h. Viable cells were quantified using the MTT assay. All values are mean ± SD (*n* = 3).

To investigate the anti-inflammatory and anti-angiogenic activities of PEDF34-NP under hypoxia in cultured cells, we encapsulated the PEDF34mer peptide into PLGA nanoparticles (PLGA34-NP). Primary BRCEC and ARPE19 (i.e., cell line derived from human retinal pigment epithelium) were both treated with PEDF34-NP for 16 h under hypoxia (1% O_2_). Hypoxia-induced VEGF protein expression, as detected *via* western blot analysis, was significantly attenuated in both ARPE19 and BRCEC cells when treated with PEDF34-NP (Figures 1A,B), as compared to Control-NP treated and untreated cells. Furthermore, PEDF34-NP reduced hypoxia-induced VEGF secretion in a concentration-dependent manner as measured by ELISA (Figure 1C). Similarly, real-time RT-PCR analysis showed that PEDF34-NP, but not the Control-NP, significantly suppressed *Vegf* mRNA expression under hypoxic conditions (Figure 1D). To evaluate the inhibitory effect of PEDF34-NP on the proliferation of endothelial cells, we treated BRCEC with PEDF34-NP for 48 h and quantified the number of viable cells. As shown by the MTT assay, PEDF34-NP significantly decreased hypoxia-induced BRCEC growth in a concentration-dependent manner (Figure 1E). Under the same conditions, PEDF34-NP had no effect on ARPE19 cell viability (Figure 1F), suggesting that the inhibitory effect is endothelial cell (EC)-specific.

### 3.2 Inhibitory effect of PEDF34-NP on neovascularization in the oxygen-induced retinopathy model

**FIGURE 2:**
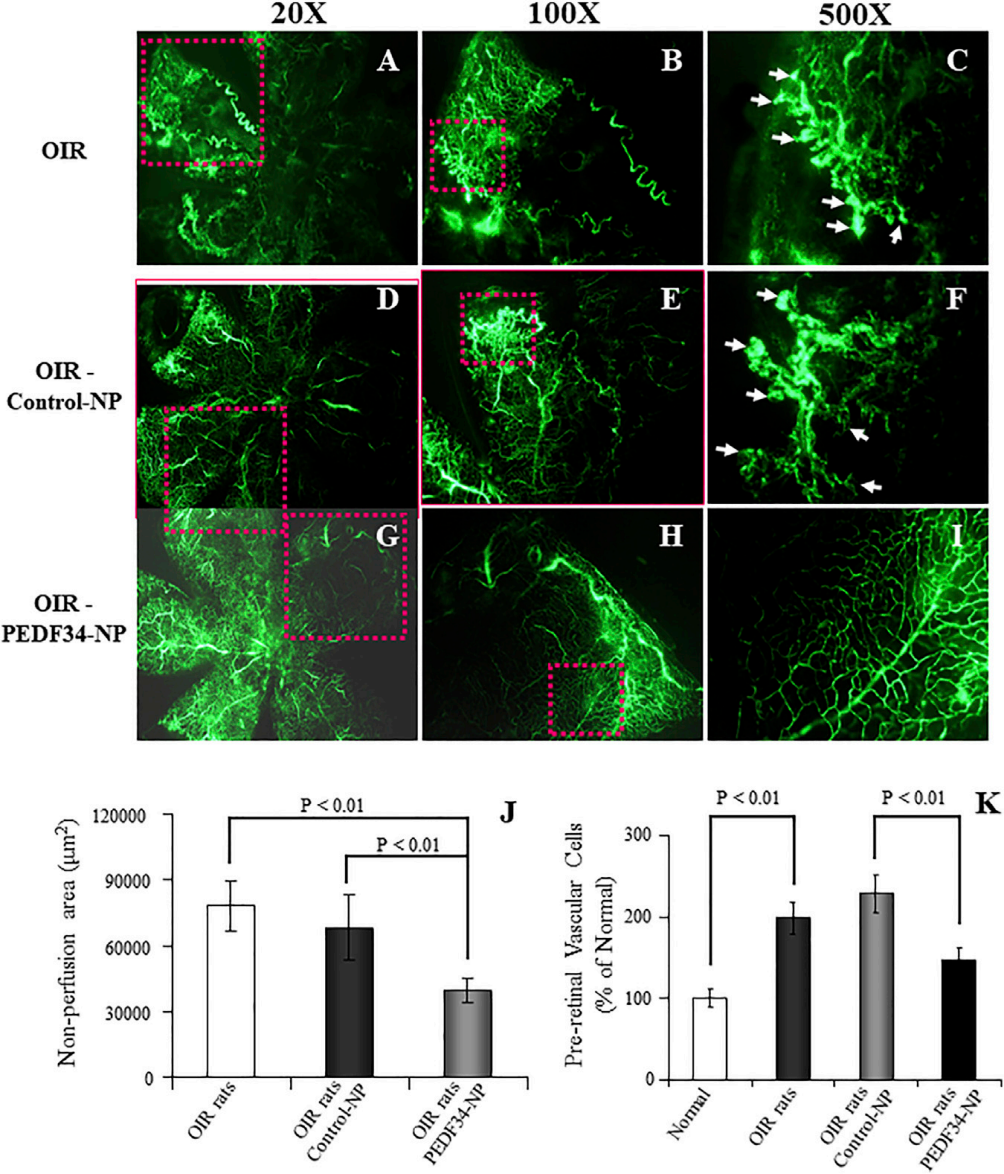
Inhibitory effects of PEDF34-NP on ischemia-induced retinal neovascularization. Rats were exposed to 75% ± 2% oxygen from P7 to P12. Retinal vasculature was examined using fluorescein angiography at P18 as described in Methods. **(A–C)** OIR rats without treatment; **(D–F)** OIR rats injected with Control-NP (5 µg/eye); **(G–I)** OIR rats injected with PEDF34-NP into the vitreous (5 µg/eye) at P12. **(J)** Non-perfusion area was measured and expressed as percent of the total retina area (mean ± SD, *n* = 6). **(K)** At P18, the eyes were fixed, sectioned and stained with hematoxylin and eosin (H&E). Pre-retinal vascular cells were counted in eight non-continuous sections per eye and averaged as described in Methods (mean ± SD, *n* = 6).

To evaluate the effect of PEDF34-NP on ischemia-induced retinal neovascularization in OIR rats, PEDF34-NP or Control-NP were injected into the vitreous at postnatal day 12 (P12). Retinal vasculature was visualized in the retinal whole-mount after FITC-dextran angiography at P18. The OIR rat retinas with the PEDF34-NP injection showed decreased neovascular tufts (Figures 2G–I) as compared to those injected with PLGA-NP control (Figures 2D–F) and untreated OIR retinas (Figures 2A–C). As the area of non-perfusion in the retina correlates with the severity of retinopathy in the OIR model, we quantified and compared the central non-perfusion area. The results showed a significantly smaller central non-perfusion area in the retinas of the OIR rats treated with PEDF34-NP, compared with OIR rats treated with the Control-NP and untreated OIR rats (Figure 2J).

As pre-retinal neovascularization is a characteristic of the OIR model, we quantified pre-retinal neovascularization by counting vascular cells extending beyond the inner limiting membrane in cross sections of the eyes. At P18, the average number of pre-retinal nuclei significantly decreased in the retina of the OIR rats treated with PEDF34-NP, compared with those with Control-NP treatment and untreated OIR rats (Figure 2K).

### 3.3 Effect of PEDF34-NP on retinal inflammation and retinal vascular leakage in oxygen-induced retinopathy rats

**FIGURE 3:**
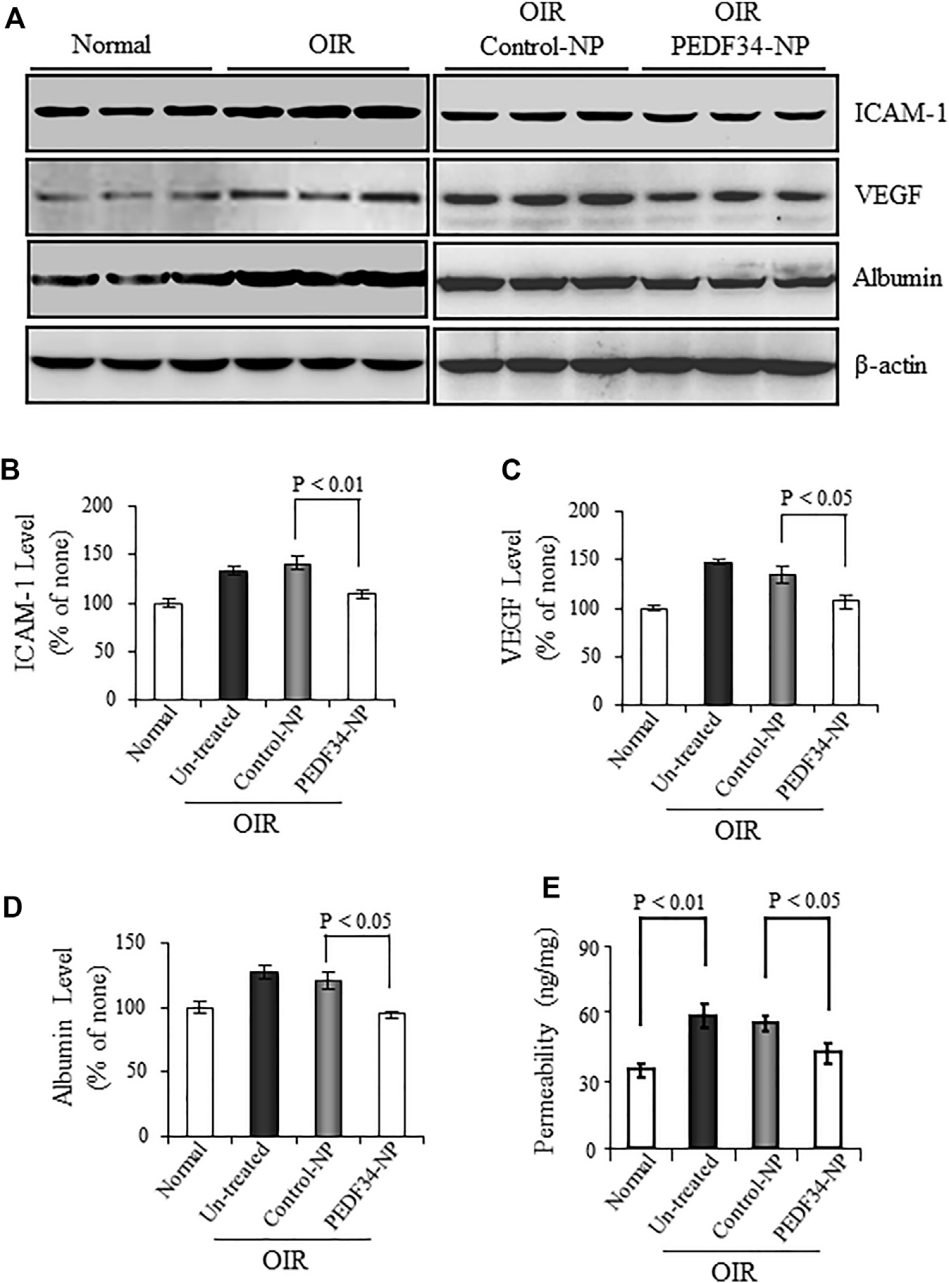
Effect of PEDF34-NP on retinal inflammatory factor expression and vascular leakage in OIR rats. OIR rats received an intravitreal injection of 5 µg PEDF34-NP or Control-NP. **(A)** At P16 the retina was perfused, and retinal levels of ICAM-1, VEGF, and albumin were determined by western blot analysis, with β-actin as loading control. **(B–D)** Retinal levels of ICAM-1, VEGF, and albumin were quantified by densitometry and expressed as % of that in normal rat retinas (mean ± SD, *n* = 3). **(E)** Retinal vascular permeability was measured using the Evans blue as tracer at P16, and normalized by the total retinal protein concentration (mean ± SD, *n* = 6).

VEGF and ICAM-1 are both well-known inflammatory factors contributing to the blood-retinal barrier (BRB) breakdown and neovascularization in OIR rats. We measured VEGF and ICAM-1 expression in the retina of these animals treated with PEDF34-NP or Control-NP. As determined by western blot analysis, VEGF and ICAM-1 levels were elevated in the retinas of OIR rats at P18, compared with the age-matched normal controls (Figure 3A). Treatment with PEDF34-NP significantly reduced the ischemia-induced retinal VEGF (27%, *p* < 0.05) and ICAM-1 (31%, *p* < 0.01) in OIR rat retinas, compared with those of retina with Control-NP injection (Figures 3B,C).

Increased retinal vascular leakage or the blood-retinal barrier breakdown is a common pathological change in DR. To evaluate the effect of PEDF34-NP on the ischemia-induced vascular leakage, we measured albumin in the perfused retina. Western blot analysis showed that PEDF34-NP significantly decreased retinal albumin levels by 30% in OIR rats, compared with that in the retina injected with Control-NP and in untreated OIR rats (Figure 3A).

To further confirm that PEDF34-NP reduce retinal vascular leakage, retinal vascular permeability was measured at P16 using the Evans blue-albumin as tracer. The PEDF34-NP injection significantly reduced vascular permeability in OIR rats, compared with those with Control-NP as well as untreated OIR rats (Figure 3E). No difference in the retinal vascular leakage was observed between the eyes injected with Control-NP and untreated OIR rats (Figure 3E).

### 3.4 Effect of PEDF34-NP on retinal vascular leakage and high-expression of inflammatory and fibrogenic factors in the retina of streptozotocin -induced diabetic rats

**FIGURE 4:**
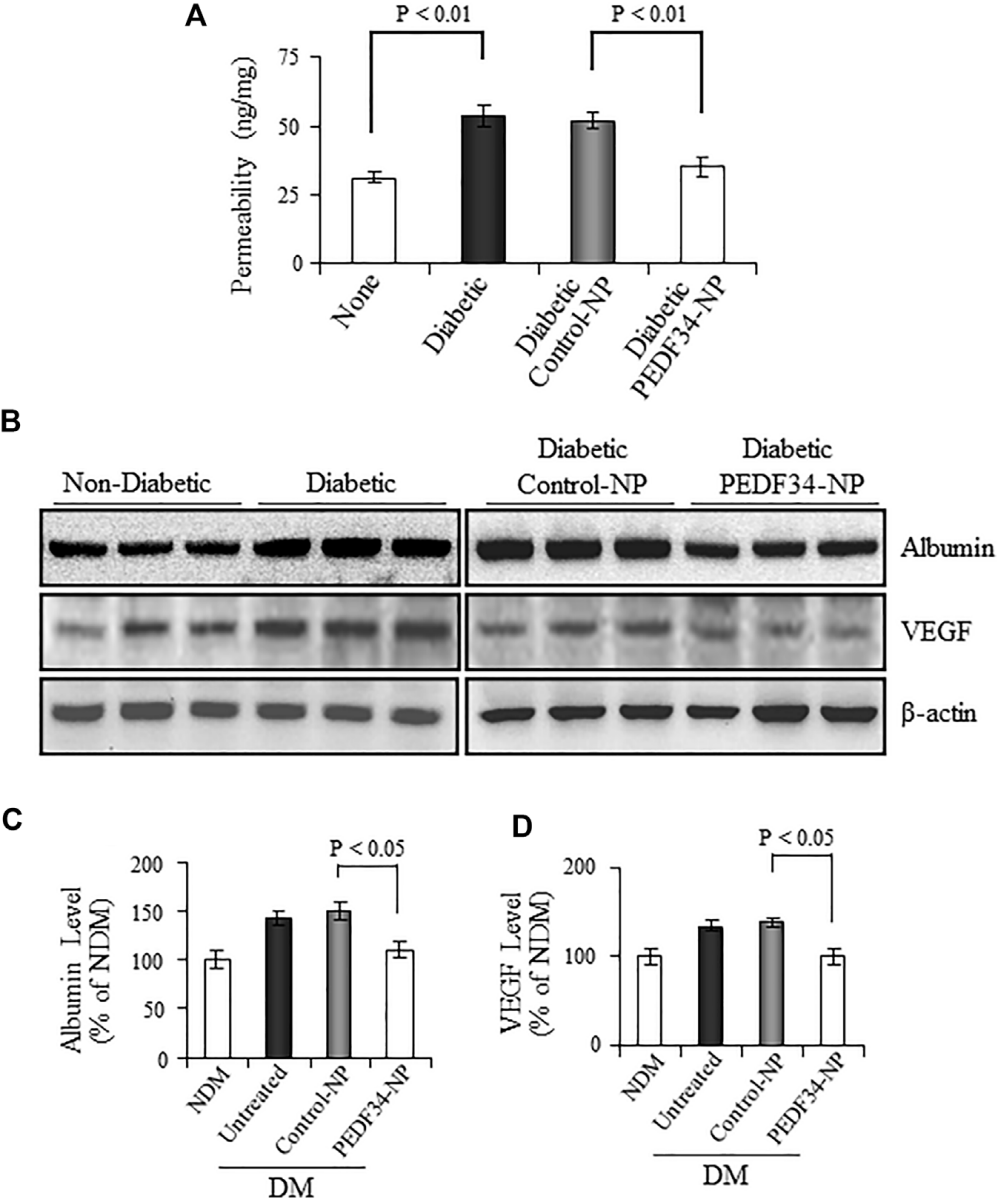
Effect of PEDF34-NP on retinal vascular leakage in STZ-induced diabetic rats. Diabetic rats received an intravitreal injection of 5 μg PEDF34-NP or Control-NP. **(A)** Four weeks after the injection, retinal vascular permeability was measured using Evans blue as tracer and normalized by total retinal protein concentration (mean ± SD, n = 5). **(B–D)** Retinas were dissected after perfusion, and the levels of albumin and VEGF were determined by western blot analysis, normalized to β-actin levels, semi-quantified by densitometry, and expressed as % of those in non-diabetic rat retinas (mean ± SD, *n* = 3).

To evaluate the effects of PEDF34-NP on DR, we injected PEDF34-NP into the vitreous of STZ-induced diabetic rats, at 2 weeks after the onset of diabetes. We first measured vascular permeability using the Evan blue-albumin leakage method at 4 weeks after the injection. Compared with non-diabetic rats, retinal vascular permeability was significantly increased in un-treated diabetic rats (*p* < 0.01) and diabetic rats treated with Control-NP (*p* < 0.01) (Figure 4A). PEDF34-NP injection resulted in a significant reduction of retinal vascular leakage in STZ-induced diabetic rats, relative to the diabetic rats treated with Control-NP (*p* < 0.01) (Figure 4A). Western blot analysis showed that albumin levels in the perfused retinas were significantly increased in diabetic rats (Figures 4B,C). PEDF34-NP significantly suppressed the albumin leakage into the retina in diabetic rats, compared to un-treated diabetic rats and those treated with Control-NP (*p* < 0.05) (Figures 4B,C). In contrast, no significant differences were detected in the retinal albumin levels between untreated diabetic rats and diabetic rats treated with Control-NP (Figures 4B,C).

VEGF is a major permeability factor that contributes to vascular leakage in diabetic retinas. We determined the effect of PEDF34-NP on VEGF protein expression and found that it was highly expressed in the retinas of diabetic rats, and was significantly downregulated by treatment with PEDF34-NP (Figures 4B,D).

**FIGURE 5:**
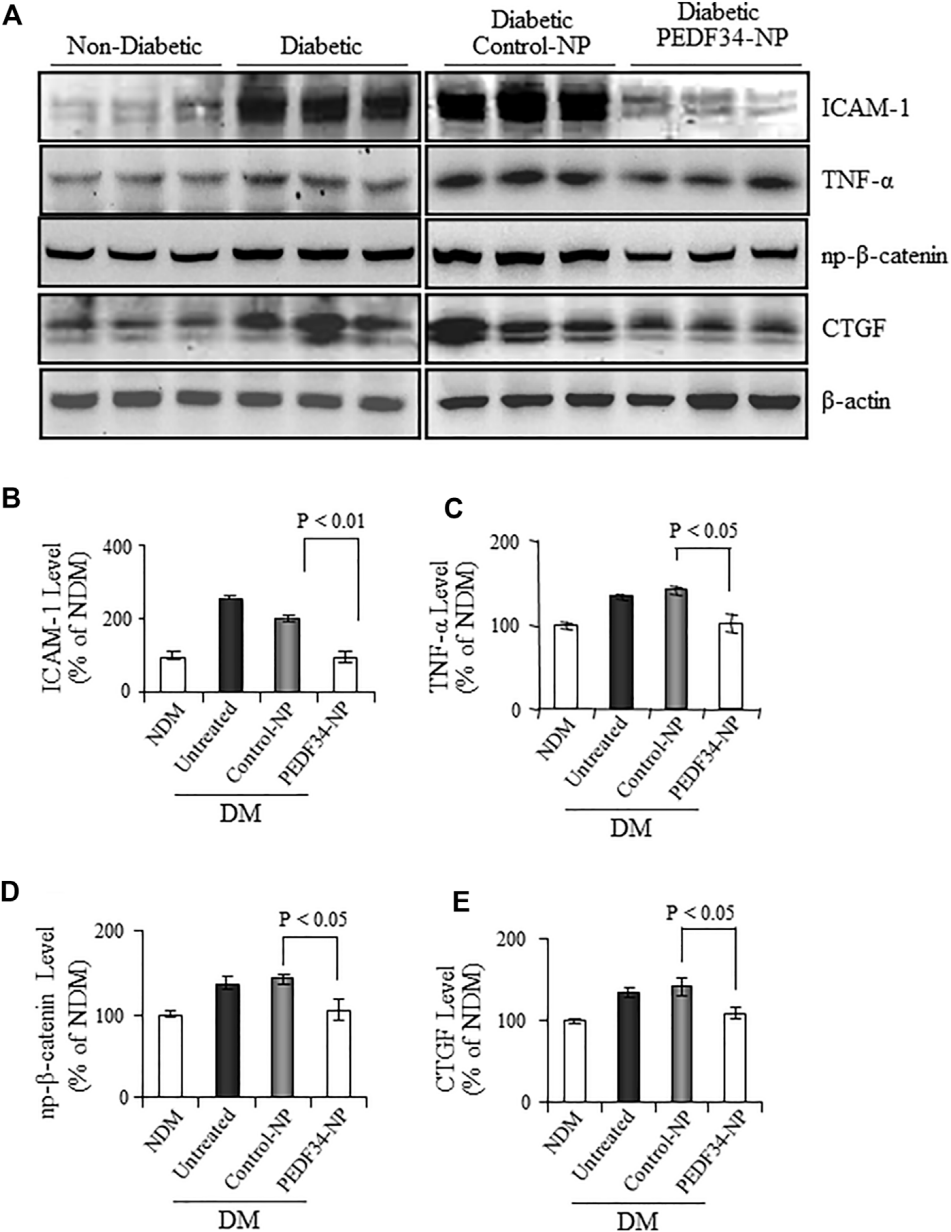
PPEDF34-NP decreased retinal levels of non-phosphorylated β-catenin (NP-β-catenin), inflammatory, and fibrogenic factors in STZ-induced diabetic rats. Diabetic rats received an intravitreal injection of 5 μg of PEDF34-NP or Control-NP. **(A)** Four weeks after the injection, retinal levels of ICAM-1, TNF-α NP-β-catenin, and CTGF were determined by western blot analysis. PEDF34-NP significantly reduced the levels of ICAM-1 **(B)**, TNF-α **(C)**, NP-β-catenin **(D)**, and CTGF **(E)** in the retinal tissue. The proteins levels were semi-quantified by densitometry, normalized by β-actin levels, and expressed as % of those in non-diabetic rat retinas (mean ± SD, *n*=3).

We next examined the anti-inflammatory effects of PEDF34-NP in diabetic rats. As shown by western blot analysis, retinal levels of the inflammatory factors TNF-α and ICAM-1 were significantly elevated in diabetic rats (Figure 5A), and the increase was attenuated in diabetic rats 4 weeks after PEDF34-NP injection. The expression of both TNF-α (*p* < 0.05) and ICAM-1 (*p* < 0.01) was suppressed by PEDF34-NP injection relative to diabetic rats injected with Control-NP (Figures 5A–C). Our previous studies have shown that activation of the canonical Wnt pathway in the retina plays a key role in retinal inflammation in DR (Chen et al., 2009; Liu et al., 2013). We examined Wnt signaling activity by western blot analysis of β-catenin. The results showed that non-phosphorylated β-catenin increased in the retina of diabetic rats, while PEDF34-NP significantly attenuated the increases of non-phosphorylated β-catenin in the diabetic retina (Figures 5A,D). CTGF is a Wnt signaling target gene and fibrogenic factor involved in the thickening of the capillary basement membrane in DR. Western blot analysis showed that CTGF expression was upregulated in the retina of diabetic rats, which was attenuated by PEDF34-NP (Figures 5A,E). These results suggest a role of PEDF34-NP in modulating Wnt signaling in order to regulate vessel permeability, inflammation, and fibrosis in diabetic retinas.

**FIGURE 6:**
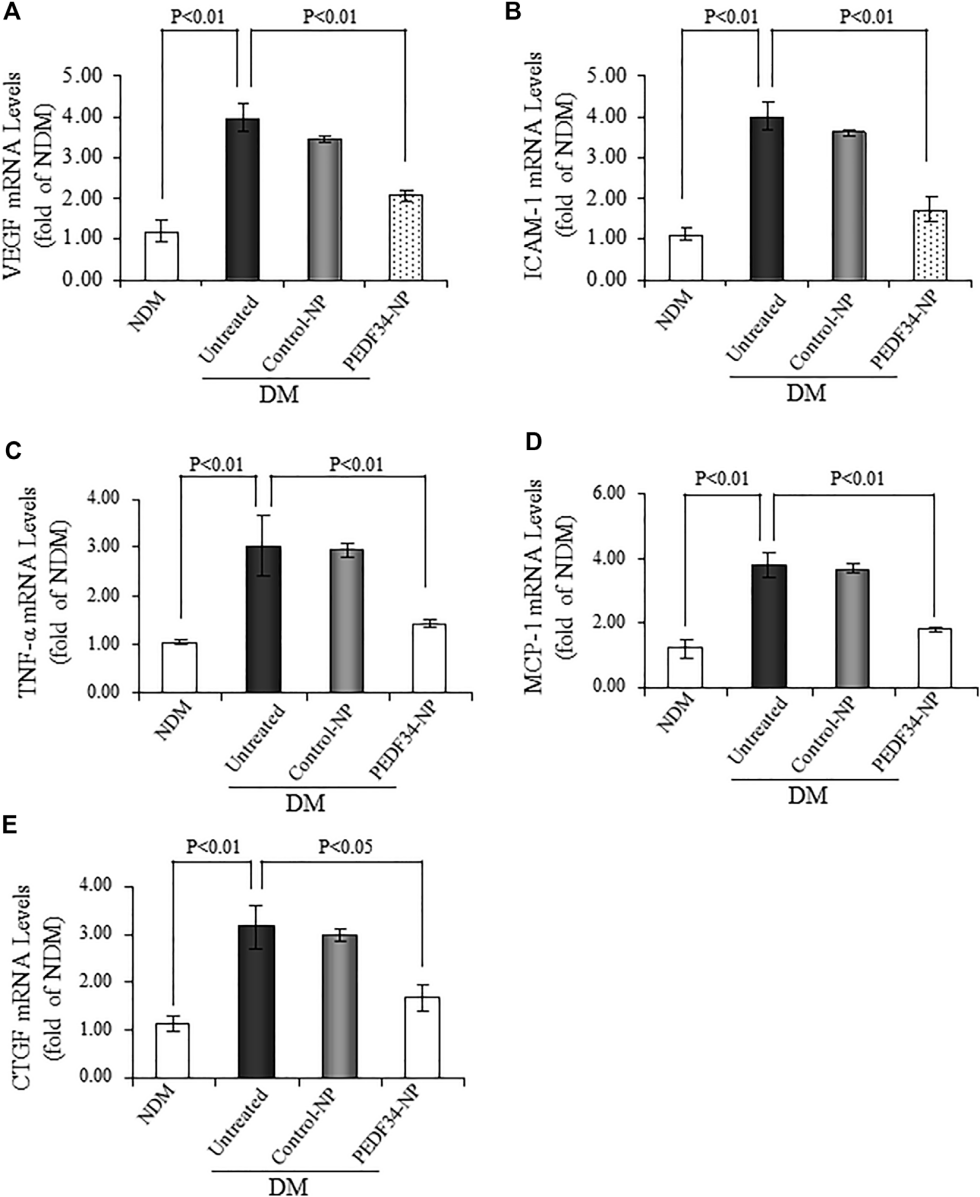
PEDF34-NP treatment decreased mRNA levels of inflammatory and fibrogenic factors in the STZ-induced diabetic rats. Diabetic rats received an intravitreal injection of 5 μg of PEDF34-NP or Control-NP. RNA was extracted from the retina 4 weeks after the injection, and mRNA levels of *Vegf* **(A)** *, Icam-1* **(B)** *, Tnf-α* **(C)**, *Mcp-1* **(D)**, and *Ctgf* **(E)** were semi-quantified by real-time RT-PCR and expressed as fold of that in non-diabetic rat retinas (mean ± SD, *n* = 3).

To confirm the inhibitory effect of PEDF34-NP on the upregulated expression of the inflammatory and fibrotic factors discussed above, we measured the mRNA levels of *Vegf*, *Icam-1*, *Tnf-α*, *monocyte chemoattractant protein-1* (*Mcp-1*), and *Ctgf* in the retina using real-time RT-PCR, 2 weeks after the injection of PEDF34-NP. The mRNA levels of these factors were downregulated by PEDF34-NP (*p* < 0.01) treatment, but not by Control-NP, in the retina of STZ-diabetic rats (Figures 6A–E).

### 3.5 Lack of detectable ocular toxicities of PEDF34-NP

**FIGURE 7:**
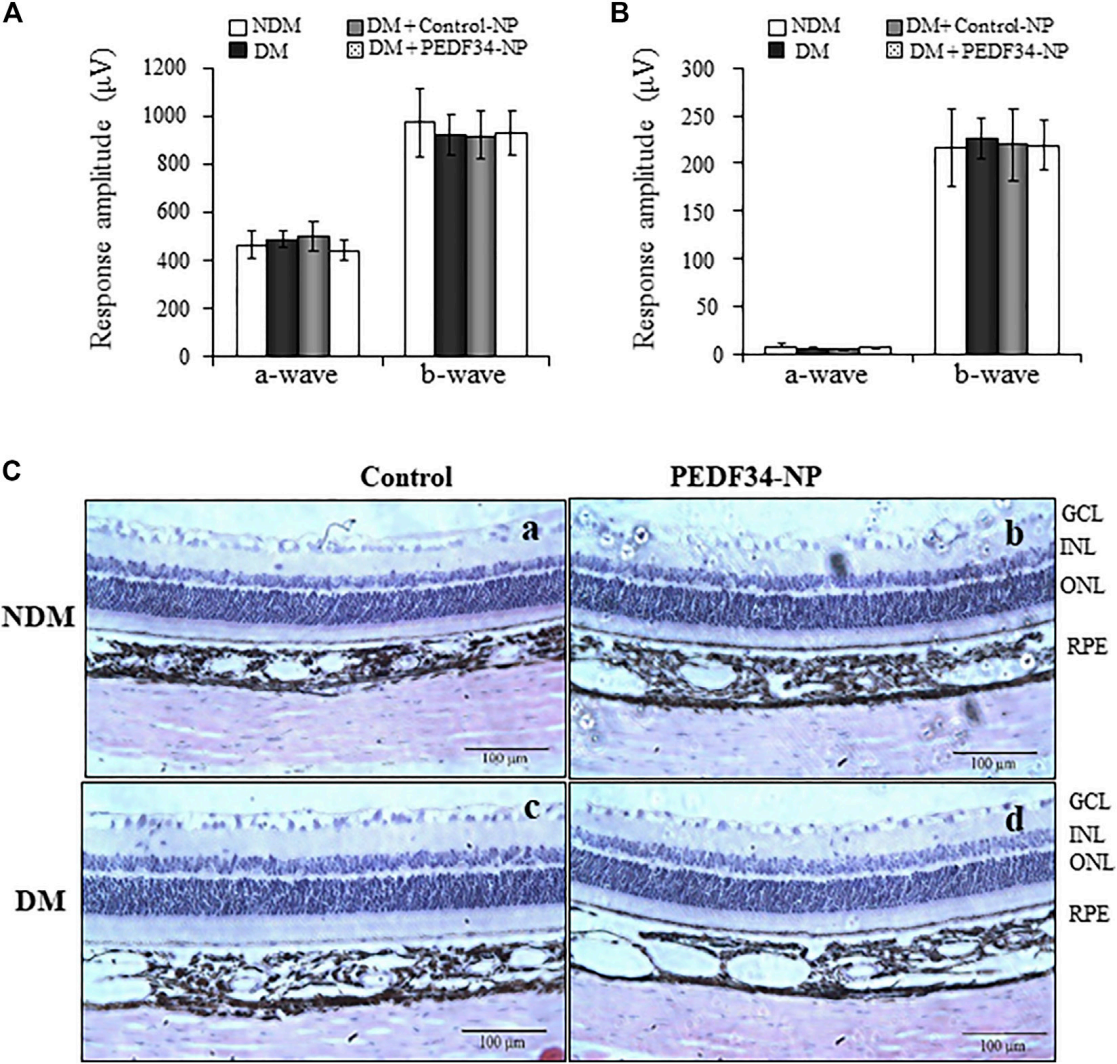
No changes in ERG response and retinal histology in response to PEDF34-NP injection. Scotopic and photopic ERG were recorded from six rats at 4 weeks after the intravitreal injection of PEDF34-NP and Control-NP as indicated. Amplitudes of a- and b-waves from scotopic **(A)** and photopic **(B)** ERG were averaged and compared (mean ± SD, *n* = 6). **(C)** Representative images of retinal section stained with H&E from non-diabetic rats **(A)**, non-diabetic rats (NDM) injected with PEDF34-NP **(B)**, diabetic rats (DM) **(C)**, and diabetic rats injected with PEDF34-NP **(D)**.

To determine possible ocular toxicities of PEDF34-NP *in vivo*, we injected PEDF34-NP or Control-NP into the vitreous of non-diabetic rats and STZ-induced diabetic rats. Visual function was evaluated by electroretinogram (ERG) recording at 4 weeks after injection. As shown by ERG, there was no significant difference in a- or b-wave amplitudes between untreated eyes and the eyes injected with Control-NP or PEDF34-NP in diabetic and non-diabetic rats (Figures 7A,B). In addition, we examined possible toxicities of NPs to the retinal structure using histological examination of ocular cross sections. No differences were detected in the retinal layers, structure or thickness between un-treated rats and in the retinas 4 weeks post injection of PEDF34-NP (Figure 7C). Taken together, these results suggest that there were no detectable ocular toxicities after the PEDF34-NP treatment.

## 4 Discussion

PEDF is a major angiogenic inhibitor in the eye that has been shown to regulate inflammation, vascular permeability, pathological angiogenesis, and fibrosis (Dawson et al., 1999; Bouck, 2002; Tombran-Tink and Barnstable, 2003a; Barnstable and Tombran-Tink, 2004). Our previous studies reported that an disturbed balance between VEGF and PEDF expression in the retina plays a major role in the pathogenesis of DR (Gao et al., 2001). PEDF-deficient mice demonstrated higher VEGF expression and severe retinal neovascularization, compared to wild-type (Wt) mice with OIR (Dawson et al., 1999; Huang et al., 2008). Moreover, PEDF transgenic mice overexpressing PEDF showed alleviated ischemia-induced neovascularization and vascular leakage in OIR and laser-induced choroidal neovascularization (Park et al., 2012). Most strikingly, a previous study showed that the N-terminal region of PEDF (residues 44–77) possesses the anti-angiogenic activities, suggesting that the short peptide may be sufficient to achieve therapeutic effect of PEDF (Filleur et al., 2005; Longeras et al., 2012). However, the naked proteins and peptides have a short half-life (<48 h) in the retina after intravitreal injection (Zhang et al., 2001) and would require repetitive injections in order to achieve therapeutic effects. To avoid frequent, repetitive injections, we encapsulated the PEDF34 peptide into biodegradable nanoparticles in an attempt to lengthen its half-life *in vivo*. A single intravitreal injection of PEDF34-NP exhibited therapeutic effects on inflammation and vascular leakage for at least 4 weeks in a DR model, suggesting sustained release of the PEDF34 peptide. Further, PEDF34-NP also showed beneficial effects on ischemia-induced retinal neovascularization and fibrosis in the retina. These findings suggest that nanoparticle-mediated delivery of an endogenous anti-angiogenic peptide has therapeutic potential for DR and retinal neovascularization.

DR is a chronic inflammatory disorder (Klein, 1996; Aiello et al., 1998). Upregulated expression of inflammatory factors such as TNF-α, MCP-1 and ICAM-1 plays a causative role in retinal inflammation (Tang and Kern, 2011). In our DR model, expression of these inflammatory factors was increased in the retina at both mRNA and protein levels, and the increases were attenuated by PEDF34-NP injection, demonstrating that this PEDF34 peptide has anti-inflammatory activities.

Vascular leakage or breakdown of the blood-retinal barrier in the retina can lead to DME, which is the most common cause for vision loss in diabetic patients (Aiello et al., 1998; Antonetti et al., 1998). In the DR model, injection of PEDF34-NP significantly reduced retinal vascular leakage, as shown by measurement of retinal albumin levels and the Evans blue-albumin leakage assay. VEGF is a key permeability factor and plays an important role in retinal vascular leakage in DR (Adamis et al., 1994; Aiello et al., 1997). Our results demonstrated that PEDF34-NP attenuated the expression of VEGF in the retinas of both the DR and OIR models, which may be responsible for the reduction of retinal vascular leakage and neovascularization in our models.

Most diabetic rodent models do not develop retinal inflammation-associated neovascularization as is found in PDR patients (Engerman and Kern, 1995). OIR, although a non-diabetic model, is commonly used for studying ischemia-induced retinal neovascularization, which develops many pathological features, such as pre-retinal neovascularization, vascular leakage and high expression of VEGF, recapitulating the pathologies found in PDR (Smith et al., 1994). Here we found that PEDF34-NP significantly alleviated ischemia-induced retinal neovascularization, correlating with attenuation of VEGF overexpression. Consistently, PEDF34-NP also inhibited EC proliferation in culture of BRCEC under hypoxia. These observations support an anti-angiogenic activity of PEDF34-NP. In addition, PEDF34-NP also attenuated high-expression of CTGF, a fibrotic factor, in diabetic retina, suggesting that the PEDF34 peptide also retains the anti-fibrotic activity of the full-length PEDF protein.

PEDF has broad beneficial functions including anti-inflammation, anti-pathological angiogenesis and anti-fibrosis (Dawson et al., 1999; Bouck, 2002; Tombran-Tink and Barnstable, 2003a; Barnstable and Tombran-Tink, 2004) without a single receptor that can mediate these functions. We found that PEDF binds to LRP6, a major co-receptor of the Wnt signaling pathway and can antagonize Wnt signaling which is known to mediate inflammation, pathological angiogenesis and fibrosis through upregulation of TNF-α, ICAM-1, VEGF and CTGF expression in the retinas of both the OIR and DR models (Park et al., 2012). Our previous study has shown that blockage of Wnt signaling has therapeutic effects on DR (Lee et al., 2012). Thus, antagonizing Wnt signaling may represent a mechanism for the anti-inflammation, anti-angiogenesis and anti-fibrosis functions of PEDF. The present study demonstrated that PEDF34-NP attenuated the increase of np-β-catenin, an essential effector of the canonical Wnt signaling pathway, in diabetic retina. This finding demonstrates that the PEDF34 peptide retained the Wnt inhibitory activity of the intact PEDF protein.

There are three main systems of local administration of retina-protective proteins (Rebustini et al., 2021): 1), cell transplantation: the retina-protective proteins are delivered through the grafted cells *in vitro via* DNA expression vectors packaged into viral and non-viral carriers, to produce recombinant proteins. These infected or transfected cells, exemplified by RPE or induced pluripotent stem cells, are then injected into the retina; 2), gene delivery: the protein delivery is achieved with DNA expression vectors packaged into viral and non-viral carriers to prompt the expression of the proteins in the retina; 3), pharmacologic delivery: the purified recombinant proteins or synthetic peptides are packaged into different types of carriers (such as micro-particles/-emulsions or nanoparticles) and introduced into the vitreous. In addition, there are four routes of delivery retina-protective proteins, including intravitreal injection, subretinal injection, subconjunctiva injection, and the use of topical eye drops. Previous studies demonstrated that surface-modified PLGA-NPs were used for topical ocular drug delivery to treat the acquired retinal vasculopathies (Dandamudi et al., 2021); PEDF gene loaded in PLGA–NP significantly inhibited *in vitro* CT26s (colon carcinoma cell line) proliferation and the growth of colon carcinoma in an *in vivo* model by inducing cell apoptosis and inhibiting angiogenesis (Cui et al., 2010). However, no documented study has reported the constant release of PEDF peptide encapsulated in PLGA-NP for the prevention or treatment DR *via* intra-vitreous injection.

Intravitreal drug delivery is required for the treatment of retinal disorders due to the blood-retinal barrier. Our previous study demonstrated that the effect of a small anti-angiogenic peptide such as kringle 5 (K5), disappeared from the vitreous and retina at 3–4 days after intravitreal injection (Zhang et al., 2001). Frequent intravitreal injections are associated with high costs and risks for long-term treatment. As such, a sustained drug release system is desired for the long-term treatment of chronic ocular diseases. Nanoparticle-mediated drug delivery is a promising method to achieve sustained intraocular release of drugs in ocular tissues. PLGA-based nanoparticles are biodegradable without severe toxicities (Park et al., 2009). The US Food and Drug Agency has approved PLGA for the use in many long-acting drug formulations. As such, we encapsulated the PEDF34 peptide into PLGA nanoparticles to achieve sustained release in the vitreous. In this proof-of-concept study, our results showed that the biological effect of the PEDF34 peptide can be sustained for at least 4 weeks after a single PEDF34-NP injection, suggesting that the bio-active peptide is present in the vitreous and retina for a prolonged time. A limitation of this study is that we only measured the therapeutic effect of PEDF34-NP for 4 weeks. As DR required long-term treatment, in the future, the effects of PEDF34-NP for further prolonged periods after a single injection needs to be evaluated.

To exclude cytotoxicity associated with nanoparticle-mediated small peptides (Seyfoddin et al., 2010), we also evaluated ocular toxicities of PEDF34-NP. Our *in vitro* studies did not show any cytotoxicity of PEDF34-NP. In addition, our *in vivo* data showed no alternations in retinal structure and function after PEDF34-NP injection. As shown by ERG recording, retina function is unchanged after the injection, and no histo-pathologies associated with the injection were detected, demonstrating the safety of the nano-particle-mediated peptide delivery.

## 5 Conclusion

This study demonstrated that an intravitreal injection of an anti-antiangiogenic peptide of PEDF encapsulated with PLGA polymer displayed potent effects on retinal inflammation, vascular leakage, and neovascularization over 4 weeks after a single intravitreal injection. This nanoparticle-mediated sustained release of the PEDF34 peptide has potential to become a therapeutic strategy for DME and retinal neovascularization associated with other ocular disorders such as PDR, and other diabetic complications.

## Data Availability

The original contributions presented in the study are included in the article/Supplementary material, further inquiries can be directed to the corresponding author.
