# Supplementary material for: Sustained therapeutic effect of an anti-inflammatory peptide encapsulated in nanoparticles on ocular vascular leakage in diabetic retinopathy
Source: Front Cell Dev Biol. 2022 Dec 16;10:1049678. doi: 10.3389/fcell.2022.1049678 (PMC9802579; doi:10.3389/fcell.2022.1049678)
Supplement: Supplementary file 1 [file Table1.DOCX]

**Table S1. The list of primers used in this study.**

| **Primers**  VEGF forward | 5′-ATGAACTTTCTGCTGTCTTGGGTG-3′ |
| --- | --- |
| VEGF reverse | 5′-TCACCGCCTCGGCTTGTCACA-3′ |
|  |  |
| TNF-α forward | 5’-ACAAGGCTGCCCCGACTAT-3’ |
| TNF-α reverse | 5’-CTCCTGGTATGAAGTGGCAAATC-3’ |
|  |  |
| CTGF forward | 5′-GTTGTTCATTAGCGCACAGTG-3′ |
| CTGF reverse | 5′-TAGAGCAGGTCTGTCAAGCAT-3′ |
|  |  |
| GAPDH forward | 5’-ATGGTGAAGGTCGGTGTGAAC-3’ |
| GAPDH reverse | 5’-GTGCCGTTGAATTTGCCGTGA-3’ |
